# An algorithm to predict intrathecal IgM synthesis in multiple sclerosis patients: optimizing the use of oligoclonal band testing

**DOI:** 10.1515/almed-2025-0098

**Published:** 2025-12-08

**Authors:** Jordi Tortosa-Carreres, Laura Cubas-Núñez, Jéssica Castillo-Villalba, Lorena Forés-Toribio, Mónica Piqueras, Raquel Gasque-Rubio, Sara Carratalà-Boscà, Carlos Quintanilla-Bordás, David Gorriz-Romero, Bonaventura Casanova, Begoña Laiz-Marro, Francisco Carlos Pérez-Miralles

**Affiliations:** Laboratory Department, 16273 La Fe University and Polytechnic Hospital, Valencia, Spain; Neuroimmunology Research Group, Health Research Institute La Fe, Valencia, Spain; Neurology Department, General University Hospital of Castellón, Castelló, Spain; Neuroimmunology Unit, La Fe University and Polytechnic Hospital, Valencia, Spain

**Keywords:** biomarker optimization, intrathecal synthesis, multiple sclerosis, oligoclonal IgM band

## Abstract

**Objectives:**

We aimed to develop a simple, cost-effective algorithm to predict the presence of oligoclonal IgM bands (OCMB) in patients with multiple sclerosis (MS), using surrogate markers such as the IgM Index (IgMi) and the Free Kappa Light Chains Index (FKLCi), with the aim of reducing the need for OCMB testing while maintaining high diagnostic accuracy.

**Methods:**

A retrospective study was conducted using paired serum and cerebrospinal fluid samples from patients diagnosed with MS at disease onset at Hospital Universitari i Politècnic La Fe. Receiver operating characteristic (ROC) curve analyses were performed for IgMi and FKLCi to evaluate their ability to predict OCMB positivity. Several combinations of cutoff values prioritizing high specificity and positive predictive value (PPV) were tested, and the best-performing threshold combination was selected.

**Results:**

A total of 97 patients were included. The optimal model defined OCMB positivity as having either IgMi≥0.2 or FKLCi≥250. Applying this algorithm reduced the need for OCMB testing by 33 %, while achieving a specificity of 90 % and a PPV of 85 %.

**Conclusions:**

IgMi and FKLCi may serve as reliable predictors of OCMB positivity in MS, potentially reducing unnecessary testing and laboratory workload.

## Introduction

Despite ongoing advances in the prognostic validation of other biomarkers, the detection of intrathecal IgM synthesis (ITS-IgM) through IgM oligoclonal bands (OCMB) remains a widely used approach in multiple sclerosis (MS) research. OCMB positivity has been associated with greater long-term disability [1], 2], poorer treatment responses [1], 2], a higher likelihood of conversion to progressive disease forms [3], increased relapse risk [1], and, more recently, a greater tendency to exhibit ongoing disease activity [4]. Nevertheless, a definitive consensus on its clinical application remains lacking [5], 6].

Owing to the pentameric structure of IgM and its low concentrations in cerebrospinal fluid (CSF) – approximately 200-fold lower than IgG – the detection of OCMB is technically more challenging and labor-intensive than that of oligoclonal IgG bands (OCG) requiring the expertise of highly specialized professionals [6], 7]. This complexity may partially explain the limited body of literature available on this biomarker. Conversely, the prognostic value of OCG is limited by their extremely high prevalence among patients with MS (pwMS).

Building on our previous work, in which we developed an algorithm to support the detection of intrathecal IgG synthesis [7], we now sought to conduct a similar study aimed at developing a predictive algorithm for OCMB detection. Specifically, we assessed whether the IgM index (IgMi) and the free kappa light chain index (FKLCi; FKLC=free kappa light chain) could reliably predict OCMB positivity in pwMS, with the goal of reducing or potentially avoiding the need for OCMB testing.

## Materials and methods

A retrospective cross-sectional study was conducted. The inclusion criteria were based on the availability of CSF at disease onset in pwMS diagnosed according to the McDonald criteria applicable at the time of diagnosis. Samples were provided by the Neuroimmunology Unit and biobank of Hospital Universitari i Politècnic La Fe (València). The diagnosis of patients and CSF and serum sample collection were performed between 2015 and 2022.

CSF and blood samples were obtained by lumbar puncture and venipuncture using a sterile and gel separator tube respectively and immediately centrifuged. The resulting cell free CSF and serum was aliquoted in polypropylene tubes, kept at 4 °C less than 24 h and finally stored at −80 °C for further analysis.

**Figure 1:**
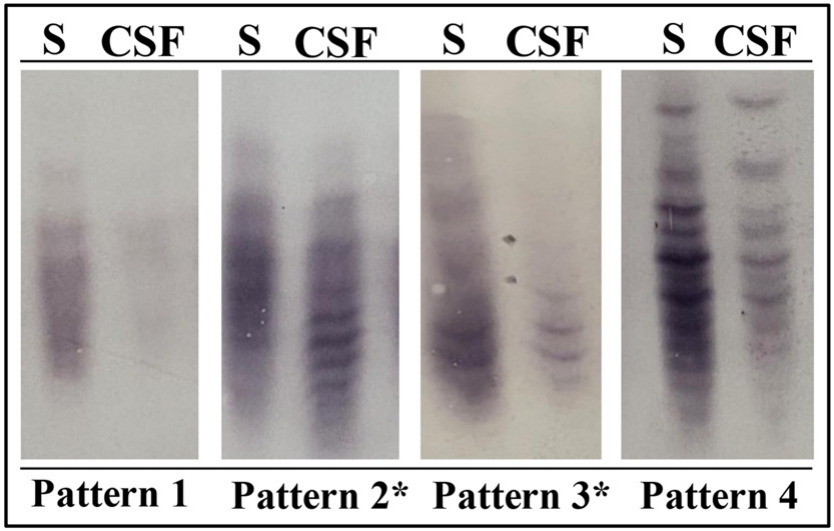
Representative examples of OCMB patterns in paired serum and CSF. Pattern 1: no bands in CSF and serum; Pattern 2: bands restricted to CSF; Pattern 3: additional CSF bands beyond those in serum; Pattern 4: identical bands in serum and CSF (‘mirror pattern’). *Patterns considered positive, as they correspond to intrathecal IgM synthesis. CSF: cerebrospinal fluid; S: serum.

Quantifications of albumin and IgM levels in serum and CSF were performed using nephelometer Image 800^®^ (Beckman Coulter, CA, USA). FKLC was determined through turbidimetry employing Optilite^®^ (The Binding Site, Birmingham, UK) equipment. OCMB were analyzed through isoelectric focusing followed by immunoblotting according with the technique described by Villar et al. [8]. A result was considered positive if it showed pattern 2 or 3, consistent with intrathecal synthesis (Figure 1). All immunoblots were independently reviewed by two experienced observers to ensure consistency and minimize interpretation bias.

The IgMi and FKLCi were expressed as the quotient of CSF-to-serum IgM or FKLC respectively and the ratio of CSF-to-serum albumin.

Initially, a comparative analysis of IgMi and FKLCi values was conducted between OCMB+ and OCMB− patients, and correlations between IgMi and FKLCi were examined.

Cutoff points for IgMi and FKLCi were selected using ROC analysis, ensuring a specificity>85 % in predicting OCMB positivity. Several predictive models were then constructed based on these thresholds: OCMB+ was considered positive if either of the two parameters exceeded the predetermined cutoff; otherwise, OCMB determination should be performed.

For each model, sensitivity, specificity, positive predictive value (PPV), and negative predictive value (NPV) were calculated. In addition, a retrospective analysis was performed to estimate the potential reduction in OCMB determinations, expressed as the Percentage Reduction of OCMB Measurements (PROM). This was defined as the proportion of cases in which IgMi or FKLCi values exceeded the pre-established cutoff relative to the total number of determinations performed.

All data were analyzed using RStudio (version 4.4.1). Data management was performed with “*tidyr*”, “*dbplyr*”, and “*dplyr*”. Graphs were created using “*ggplot2*”, “*ggpubr*”, “*ggsignif*”, and “*patchwork*”. ROC curves, cutoff determination, and diagnostic accuracy analyses were conducted with “*pROC*” (integrated with “*ggplot2*”).

## Results

A total of 97 pwMS were enrolled in the study, of whom 24 (25 %) were male and 73 (75 %) female. The median age was 35.5 years (IQR: 18). OCMB positivity was observed in 54 patients (56 %). Ninety patients were diagnosed with relapsing-remitting MS, while 7 were classified as clinically isolated syndrome (CIS). At the time of lumbar puncture, 81 pwMS (83 %) exhibited disease activity, defined as the occurrence of a clinical relapse or the presence of gadolinium-enhancing lesions within a 90-day window before or after the procedure. None of the variables followed a normal distribution (p<0.0001).

**Figure 2:**
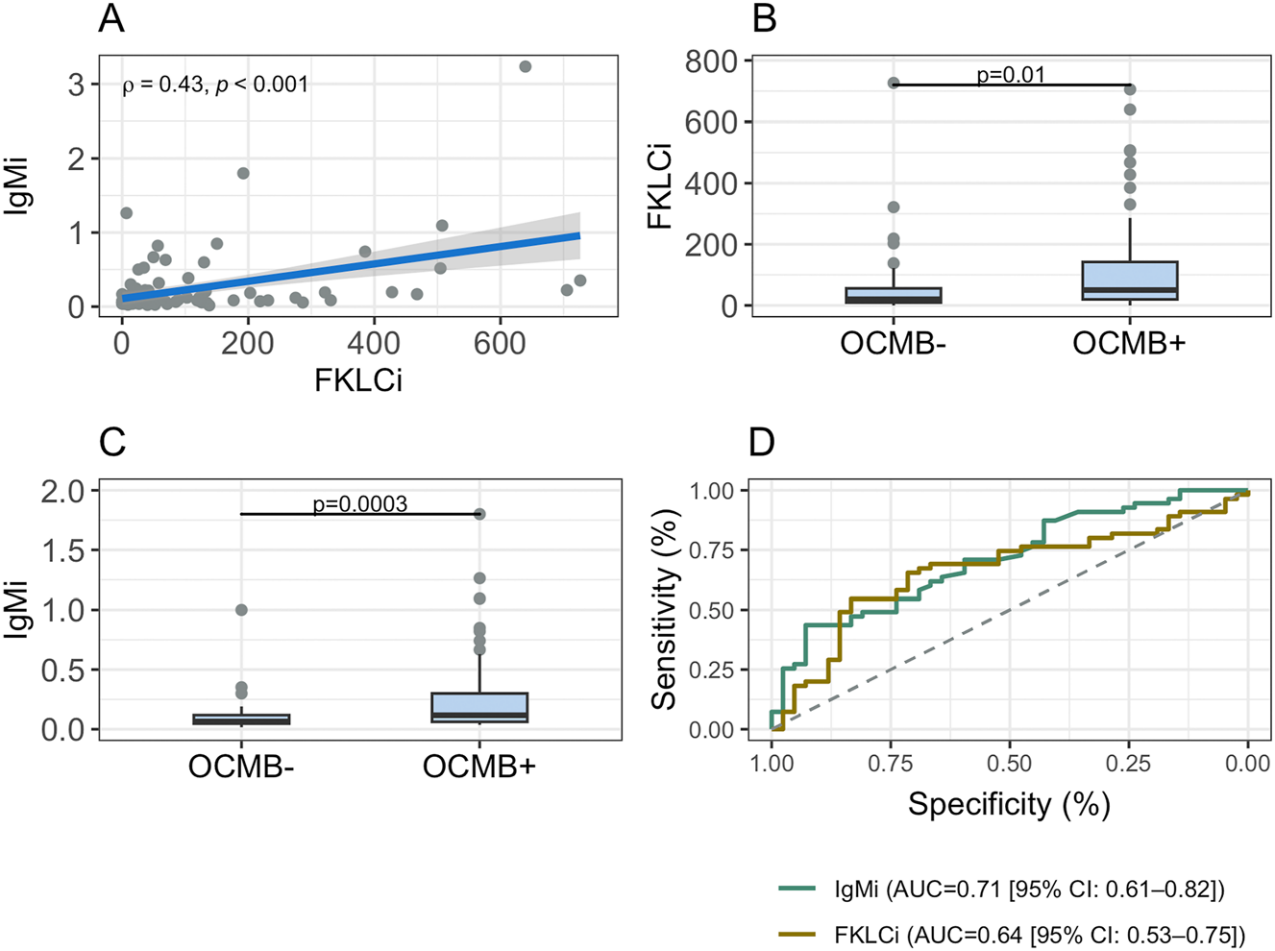
Correlation between IgMi and FKLCi (A), differences in FKLCi (B), and IgMi (C) values among patients enrolled based on the presence of OCMB. ROC curves for IgMi and FKLCi in predicting the presence of OCMB (D). FKLCi: free kappa light chain index; IgMi: IgM index; OCMB: oligoclonal IgM bands.

IgMi showed a significant correlation with FKLCi (Figure 2A). The FKLCi and IgMi of OCMB+ patients were significantly higher (Figure 2B and C). ROC curves for IgMi and FKLCi in predicting OCMB exhibited AUC of 0.71 (95%CI: 0.61–0.82) and 0.64 (95%CI: 0.53–0.75) respectively (Figure 2D). There was no difference between them.

The models were generated along with their specificities and positive predictive values, and are presented in Table 1. Model A achieved the highest PROM (36 %) (specificity=86 %; PPV=86 %); whereas Model C achieved higher specificity and PPV (90% and 88 %, respectively) at the expense of a slightly lower PROM (33 %) (Table 1), and was deemed optimal.

**Table 1:** Specificity, Sensitivity, PPV, NPV, and PROM for various thresholds utilized for IgMi and FKLCi, along with the same parameters for the different models.

	Cutoff	Specificity, %	Sensitivity, %	PPV, %	NPV, %	PROM, %
IgMi	0.2	93 (81–99)	42 (29–56)	88 (70–98)	55 (43–67)	27
IgMi	0.3	93 (81–99)	27 (16–41)	83 (59–96)	49 (38–61)	19
FKLCi	200	88 (74–96)	20 (10–33)	69 (41–89)	46 (35–57)	16
FKLCi	250	93 (81–99)	18 (9–31)	77 (46–95)	46 (35–58)	13
Model A	–	86 (71–95)	53 (39–66)	83 (66–93)	58 (45–70)	36
Model B	–	90 (77–97)	38 (25–52)	84 (64–95)	53 (41–65)	26
Model C	–	90 (77–97)	51 (37–65)	88 (71–96)	54 (41–72)	33
Model D	–	86 (71–95)	40 (27–54)	79 (59–92)	70 (55–90)	28

The thresholds for IgMi and FKLCi in the different models were as follows: Model A: 0.2/200; Model B: 0.3/250; Model C: 0.2/250; Model D: 0.3/200. Values of sensitivity, specificity, PPV, and NPV are presented along with their corresponding 95 % confidence intervals (CI). FKLCi, Free Kappa Light Chain Index; IgMi, IgM Index; NPV, negative predictive value; PPV, positive predictive value; PROM, percentage reduction in OCMB consumption.

Of note, only one CIS patient was OCMB+, and none displayed IgMi or FKLCi values≥0.2 and ≥250, respectively.

## Discussion

Based on our findings, employing an algorithm with stringent cutoff values for IgMi and FKLCi (0.2 and 250, respectively) could reduce OCMB testing by approximately one-third, while preserving high specificity and PPV. This would ease laboratory workload and accelerate turnaround times. Moreover, it could democratize access to this valuable biomarker, enabling non-specialized laboratories to benefit from ITS-IgM information using readily automatable assays such as FKLC and IgM quantification. The low sensitivity and NPV of the models are not critical limitations, as OCMB testing is still recommended for patients who do not meet the algorithmic thresholds.

In addition, our findings offer mechanistic insights. The strong correlations between IgMi and FKLCi, together with significantly elevated FKLCi values in OCMB+ patients, suggest that ITS-IgM is unlikely to be a stochastic event, but rather tends to occur in individuals with more pronounced intrathecal responses.

Among the 54 patients with OCMB positivity, 32 were correctly identified by the algorithm. While the prognostic implications of OCMB remain uncertain, this pattern suggests that patients with heightened intrathecal activity – who are also more likely to exhibit ITS-IgM – may face worse clinical outcomes. Indeed, several studies have linked high ITS-IgM levels to a more aggressive disease course [9], 10]. For example, Ozakbas et al. observed that elevated ITS-IgM was associated with spinal cord involvement, T1-hypointense lesions, and increased risk of progression to secondary progressive MS in a cohort of 85 patients [10].

Similarly, FKLCi levels exceeding 100 have been associated with earlier loss of NEDA-3 status [11], while levels above 130 were linked to earlier escalation to high-efficacy therapies [12].

Another notable observation is that all pwMS testing positive for Reibergram IgM (n=24) – a marker proposed for identifying high-risk patients [13], 14] – had IgMi values≥0.2. Consequently, IgMi was excluded from the final model to avoid redundancy.

The higher OCMB+ rate in our cohort (56 %) relative to that reported by Masi et al. (27.2 %) [4] may reflect differences in clinical context at the time of sampling. In our study, 78 % of patients exhibited active disease at diagnosis, compared to 43 % in the Masi et al. cohort [4]. Such scenario has previously been associated with elevated ITS-IgM levels [15].

Notably, the continued relevance of OCMB lies in its robust prognostic value and its association with a broader range of clinical outcomes [1], [2], [3], [4], which currently exceeds that of FKLCi [11], 12], the Reibergram [13], 14], or IgMi [9], 10]. For these alternative markers, supporting evidence remains more limited, reinforcing the need for further studies in larger and more diverse cohorts.

### Limitations

Nevertheless, this study has some limitations. First, its retrospective, single-center design may limit the generalizability of our findings, as the cohort reflects the patient population and clinical practices of our institution, which may differ from those of other centers. Second, the sample size, although suitable for preliminary modeling, may not support definitive cutoff thresholds. Third, while the algorithm correctly identified 32 of 54 OCMB+ patients, the small number of CIS cases (n=7, with only one OCMB+) precluded a reliable subgroup analysis. Thus, although the model appears robust in patients with relapsing-remitting MS, its applicability in CIS and primary progressive MS – where it has not yet been tested – remains uncertain.

Moreover, the algorithm has not yet been externally validated. Future studies including independent external cohorts are warranted to confirm the robustness of the proposed model and to specifically assess its performance in diverse clinical settings, including progressive phenotypes and CIS.

Crucially, the algorithm should be restricted to patients with confirmed intrathecal IgG synthesis – i.e., those with a high pre-test probability of MS – as it was developed in pwMS and is in this context that the test has clinical value. In addition, we recommend implementing this algorithm with laboratories defining their own optimized cutoff points, as high IgMi and FKLCi values could predict OCMB positivity and support its integration into routine practice. Further evaluation in the context of lipid-specific OCMB remains imperative.

## Conclusions

In conclusion, our algorithm may represent a pragmatic and scalable approach to reduce reliance on OCMB testing, helping to optimize laboratory resources and broaden access to prognostically relevant ITS-IgM information in clinical practice.
